# Prevalence of Vancomycin-Variable Enterococci from the Bloodstream in the Korea Global Antibiotic Resistance Surveillance System, 2017–2022

**DOI:** 10.3390/antibiotics13121210

**Published:** 2024-12-12

**Authors:** Sung Young Lee, Ji-Hyun Nam, Jung Wook Kim, Soo Hyun Kim, Jung Sik Yoo

**Affiliations:** 1 Division of Antimicrobial Resistance Research, National Institute of Health, Korea Disease Control and Prevention Agency, Cheongju-si 28159, Republic of Korea; blueskyi7@korea.kr; 2 Division of Zoonotic and Vector-Borne Disease Research, National Institute of Health, Korea Disease Control and Prevention Agency, Cheongju-si 28159, Republic of Korea; jungwookk@korea.kr; 3 Department of Laboratory Medicine, Chonnam National University Medical School, Gwangju-si 61469, Republic of Korea; alpinboy@chonnam.ac.kr

**Keywords:** global antibiotic resistance surveillance system in Korea, *vanA* gene, vancomycin susceptible, vancomycin-resistant enterococci, vancomycin-variable enterococci

## Abstract

Vancomycin-variable enterococci (VVE), though genetically containing *van* genes, are phenotypically sensitive to vancomycin. If VVE is undetected or does not grow on the vancomycin-resistant enterococci (VRE) selection medium, or both, it can acquire resistance upon exposure to vancomycin. This characteristic is clinically important for the treatment and prevention of VRE. This study aims to analyze the prevalence and characteristics of VVE in Korea through the Global Antibiotic Resistance Surveillance System (Kor-GLASS) and emphasize the importance of VVE. A total of 3342 enterococcal bloodstream isolates were collected through the Kor-GLASS between 2017 and 2022. Antibiotic susceptibility testing, *van* gene detection, and multilocus sequence typing were conducted with all the isolates. The trends in the domestic prevalence of VVE were analyzed and compared with global prevalence data. Among the isolates, 197 (5.9%), including 124 *Enterococcus faecium* and 73 *E. faecalis*, were identified as VVE. While the VRE incidence has declined in Korea since 2020, the VVE incidence is significantly rising. In Korea, only the *vanA* gene has been detected in both VRE and VVE, and no other *van* gene variants have been identified. Most of these isolates belong to CC17 (91.3%), with ST17, ST817, and ST80 as the predominant types. We have shown that continuous surveillance is essential in Korea due to the persistently high prevalence of VRE and the potential evolution of VVE into VRE. Consequently, it is critical to evaluate *Enterococcus* species isolated from domestic clinical settings for their phenotypic vancomycin resistance and the molecular detection of *van* genes, irrespective of the strain.

## 1. Introduction

Vancomycin (VAN)-resistant enterococci (VRE), whose infection usually occurs in healthcare settings, affects people who are on extended antibiotic treatment, have had surgery, have implanted medical devices, are hospitalized, or are immunocompromised. VRE was first identified in Europe in 1988 it has since emerged as a significant global health threat [1]. Namely, the prevalence of VRE infection has risen to 17.3% in Europe in 2018, and 8.7% in India in 2021. VRE infections also inflict significant mortality globally, at 60% to 70%. For instance, in the United States, VRE caused approximately 54,500 infections and an estimated 5400 deaths among hospitalized patients in 2017. In Korea, VRE was initially isolated in a tertiary hospital in 1992 [2]; its prevalence rose after 1998, reaching 30% by 2010 [3]. According to the Korea Global Antimicrobial Resistance Surveillance System (Kor-GLASS), the prevalence of blood-derived VRE further increased to 40.9% in 2019 [4]. VRE expresses genes in the *van* cluster, and can exhibit resistance to VAN. Most VRE cases in Korea involve *Enterococcus faecium*, which harbors the *vanA* gene that typically confers a high level of resistance to VAN.

Recently, VAN-variable *Enterococcus faecium* (VVEfm), which carries the *van* gene but remains susceptible to VAN under certain conditions, has been identified in several countries and regions, including Canada, Norway, Italy, Denmark, India, Bangladesh, and Australia [5,6,7,8,9,10,11]. VVE strains can phenotypically convert to vancomycin-resistant enterococci upon exposure to the antibiotic [9,12]. These strains may act as reservoirs of VAN resistance genes, increasing the risk of resistance transmission and treatment failures [6]. VVE was first reported by Szakacs et al. (2014), who confirmed the molecular characteristics of VVE isolated from clinical sources [5]. Since then, VVE strains have been continuously identified and studied. For instance, Sivertsen et al. (2016) identified a mechanism involving a silent *vanA* gene cluster capable of horizontal transfer and reverting to an active resistance state, even though antibiotic resistance to VAN was not initially confirmed [6]. This gene cluster can also be transmitted to various enterococcal species [13]. However, VVE is difficult to identify. As a result, it is often underdiagnosed, potentially posing challenges for rapid treatment and causing the global spread of VVE. Therefore, accurate identification of VVE during clinical screening is crucial for selecting the appropriate antibiotic treatment and preventing nosocomial dissemination. In addition, surveillance for these strains is important.

Surveillance of VVEfm has been reported in several countries, including Canada, Denmark, Australia, and South Korea [6,8,9,14,15]. However, most data on the prevalence of VVEfm have come from individual institutions, with limited studies conducted on a multicenter, regional, or national scale. This study aims to examine the prevalence of VVEfm at the national level in Korea by investigating and analyzing the domestic trends of VVE among all enterococcal isolates from the bloodstream infections collected through Kor-GLASS, Korea’s national antimicrobial resistance surveillance system, from 2017 to 2022.

## 2. Results and Discussion

### 2.1. Prevalence of VVE in E. faecium and E. faecalis

**Figure 1:**
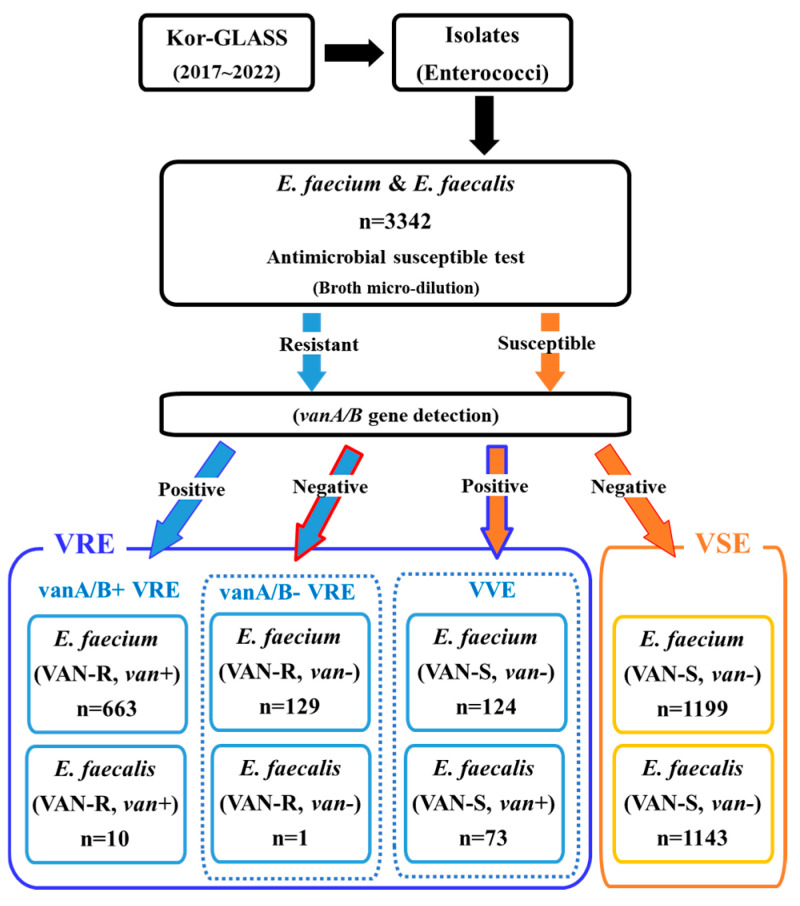
Characterization and Classification Flow Diagram of Enterococcal Isolates in This Study. vanA/B- VRE, *vanA/B* gene-negative vancomycin-resistance enterococci; vanA/B+ VRE, *vanA/B* gene-positive vancomycin-resistance enterococci; VSE, vancomycin-susceptible enterococci without the *vanA/B* gene; VVE, *vanA/B* gene-positive vancomycin-susceptible enterococci.

We collected 3342 enterococcal bloodstream isolates, of which 2115 were identified as *E. faecium* and 1227 as *E. faecalis* (Figure 1). The proportion of bloodstream enterococcal isolates among all strains identified in the Kor-GLASS program gradually increased by year, from 3.1% (464/14,781) in 2017 to 4.3% (725/16,945) in 2022. All *E. faecium* and *E. faecalis* were tested for antibiotic susceptibility, and the presence of *vanA/B* genes was investigated. These enterococci were broadly classified into VRE and VAN-susceptible enterococci without the *vanA/B* gene (VSE). We classified VRE into three distinct groups: *vanA/B*-positive vancomycin-resistant enterococci (vanA/B+ VRE), *vanA/B*-negative vancomycin-resistant enterococci (vanA/B− VRE), and *vanA/B*-positive vancomycin-susceptible enterococci (vancomycin-variable enterococci, VVE).

**Figure 2:**
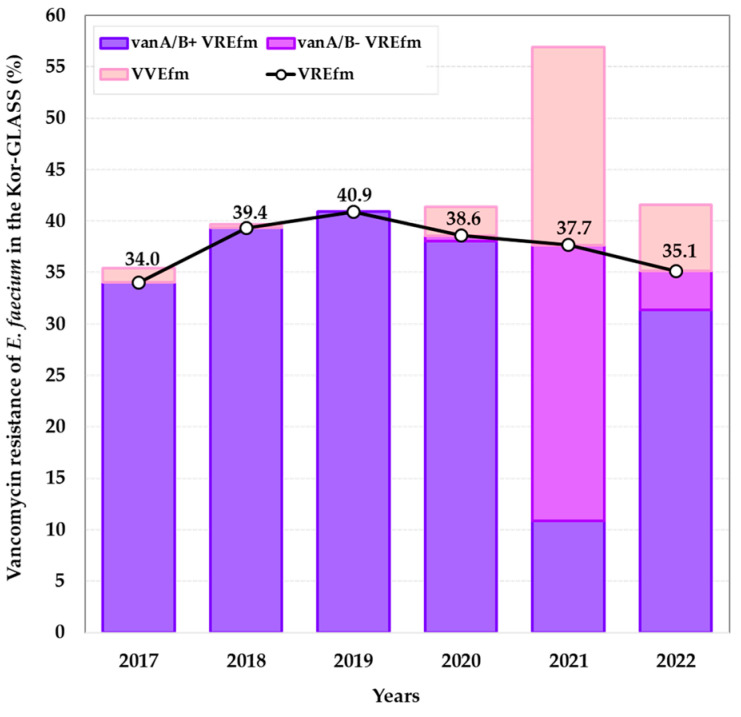
Percentage of VREfm (vanA/B+ VREfm and vanA/B− VREfm) and VVEfm from the Kor-GLASS Program in 2017 to 2022. A total of 2115 *E. faecium* were isolated from the bloodstream from 2017 to 2022. vanA/B− VREfm, *vanA/B* gene-negative vancomycin-resistant *E. faecium*; vanA/B+ VREfm, *vanA/B* gene-positive vancomycin-resistant; VVEfm, *vanA/B* gene-positive vancomycin-susceptible *E. faecium*.

The data from the Kor-GLASS program (2017 to 2022) showed that VSE was isolated at 70.1%, vanA/B+ VRE at 20.1%, vanA/B− VRE at 3.9%, and VVE at 5.9% in all enterococci (Figure 1). VRE was significantly more abundant in *E. faecium* (37.4%) than *E. faecalis* (0.9%). Among the total isolated enterococci from the bloodstream, VAN-resistant *E. faecium* gradually increased from 34.0% in 2017 to 40.9% in 2019 (Figure 2). Of all *E. faecium* isolates, 37.4% (792/2115) showed VAN resistance (VREfm). The VREfm isolates consisted of 83.7% (663/792) of vanA/B+ VREfm, carrying only the *vanA* gene, and 16.3% (129/792) of vanA/B- VREfm. The prevalence of VVEfm was 5.9% (124/2115). The prevalence of VREfm, peaking at 40.9% in 2019, has been gradually decreasing by ≥2.3% every year, except in 2021 (Figure 2). Several changes were observed during the COVID-19 pandemic from 2020 to 2021. The prevalence of vanA/B+ VREfm decreased significantly in 2021. In addition, vanA/B− VREfm and VVEfm started to increase in 2020; these changes were even more pronounced in 2021: vanA/B− VREfm and vanA/B+ VVEfm increased from 0.6% to 26.8% and from 2.8% to 19.2%, respectively. In addition, the proportion of VVEfm was significantly higher in 2021 (Figure 2).

In *E. faecalis*, the prevalence of vanA/B+ VRE was 0.8% (10/1227). In this study, all vanA/B+ VREfs isolates were confirmed to carry *vanA*. Only one of the strains was vanA/B− VREfs; it was isolated in 2021. VVE in *E. faecalis* (VVEfs) appeared in one region in 2020 and was detected sporadically in 9 areas across Korea in 2021. Although VRE and VVE were rarely reported in *E. faecalis*, the same trend was observed for *vanA*-type VVEfs (0.5% to 29.2% then to 0.4%) and *vanA*-type VVEfm (2.8% to 19.2% then to 6.4%) in 2020 to 2022 (Table S1).

In 2021, vanA/B− VRE and *vanA*-type VVE were dramatically increased, as much as vanA/B+ VRE was reduced, but did not show specific differences by month or region. These changes are probably due to the increased use of antibiotics during the COVID-19 pandemic. In particular, the daily defined dose (DDD) per 1000 inhabitants per day for glycopeptides (including VAN and TEI) increased by ≥2.8% per year during the COVID-19 pandemic (2020 to 2022) in Korea (https://nih.go.kr/nohas/statistics/selectAUStatisticsMainTab.do (accessed on 1 December 2022)). Jeon et al. (2022) reported a 49.0% increase in the prevalence of VRE in clinical samples collected from general wards during the pandemic [15]. A comparison of data from four hospitals in Korea showed that during the COVID-19 pandemic (2020–2021), vancomycin consumption increased by 16.7% in general wards but decreased by 4.0% in ICUs compared to the pre-pandemic period (2018–2019). Notably, the prevalence of VREfm in clinical samples from the wards significantly increased by 50.1%, whereas a marked 65.1% decrease was observed in surveillance samples from the ICUs. These findings suggest that weakened infection control measures and increased antibiotic usage during the pandemic contributed to the spread of VRE. The increased VAN consumption during the COVID-19 period may have facilitated a relative rise in vanA/B− VRE and VVE, while improved environmental hygiene practices may have contributed to reducing vanA/B+ VRE prevalence. However, several studies have reported that *vanA*-type VVE can convert into VRE upon exposure to glycopeptides [9,12,13], emphasizing the importance of continuous and thorough monitoring of VVE to prevent its transformation into clinically significant VRE.

### 2.2. Antimicrobial Resistance of VVE

A total of 197 VVE isolates were collected from 2017 to 2022, with 62.9% VVEfm and 37.1% VVEfs. All VVE strains exhibited susceptibility to VAN within 0.25–1.0 µg/mL MICs. On the other hand, all 673 vanA/B+ VRE isolates showed a high level of resistance to VAN above 32 µg/mL. VVEfm isolates were resistant to ampicillin (AMP; 76.9–100%), ciprofloxacin (CIP; 84.6–100%), high-level gentamycin (HL-GEN; 0–70.0%), high-level streptomycin (HL-STR; 0–10.0%), tetracycline (TET; 0–25.0%), daptomycin (DAP; 0–2.0%), and quinupristin/dalfopristin (QDA; 0–25.6%). No VVEfms exhibited resistance to teicoplanin (TEI), tigecycline (TIG), or linezolid (LIN). However, the TEI resistance rate of VREfm was observed to be 37.9% to 88.1% (Table 1). Detailed susceptibilities of VSEfm, VVEfm, and VREfm strains are shown in Table 1. Between 2020 and 2021, the QDA resistance rates of VSEfm, VVEfm, and VREfm substantially increased from 3.8% to 25.7%, 10.0% to 25.6%, and 5.1% to 52.9%, respectively (*p* < 0.001).

Among the *E. faecalis* strains, only 11 isolates were VREfs, accounting for 0.9% of the total VAN-resistant enterococci. *E. faecium* isolates exhibited high resistance to VAN, AMP, and CIP, while *E. faecalis* isolates showed high resistance to TET (Table 1 and Table S1). In 2021, we observed a sudden increase in VVEfs. Although Ayobami et al. (2020) hypothesized that the increase in VVEfs could be attributed to regional characteristics, our study did not find any significant associations between VVEfs and regional factors [16]. Like VVEfm, VVEfs did not show resistance to TEI, LIN, or TIG (Table S1). In conclusion, VVE differs from VRE in that it remains yet phenotypically susceptible to both VAN and TEI.

The *vanA*-positive strains are characterized by their inducible and high-level resistance to VAN and TEI [11,17]. Their resistance can be induced by glycopeptides, such as VAN, TEI, and avoparcin, and non-glycopeptide agents, such as bacitracin and polymyxin B [17]. After exposure to glycopeptide or similar agents, *vanA*-type VVE isolates may be converted to VAN-resistant isolates. Outbreaks of *vanA*-type VVE reversion mutations have been reported in Norway, Italy, Australia, and Korea [6,7,11,13,18]. In our study, 29.0% of VVEfm and 38.4% of VVEfs reverted to phenotypic resistance when cultured with 8 µg/mL vancomycin, which categorized them as vancomycin-resistant. Most VVE isolates (≥95.9%) reverted to vancomycin resistance when exposed to concentrations >8 µg/mL, which is consistent with previous studies in Korea [13,18]. In addition, similar reversion rates were observed at 4 µg/mL, with rates of 62.5%, 63.6%, and 96.8% in Australia, Italy, and Norway, respectively [6,7,11].

Considering the results from the Kor-GLASS program, where *vanA*-type VVE is gradually increasing, the continuous motoring of glycopeptide drugs usage and VVE appear necessary in Korea. Furthermore, ongoing surveillance of VRE strains with reduced susceptibility to LIN, TIG, and DAP, antibiotics considered last resort options for VRE treatment, is crucial to identify and prevent the emergence of LIN-resistant, TIG-resistant, and/or DAP-resistant VRE strains.

### 2.3. Comparative Analysis of VVEfm Survey Results Between Korea and Other Countries

**Figure 3:**
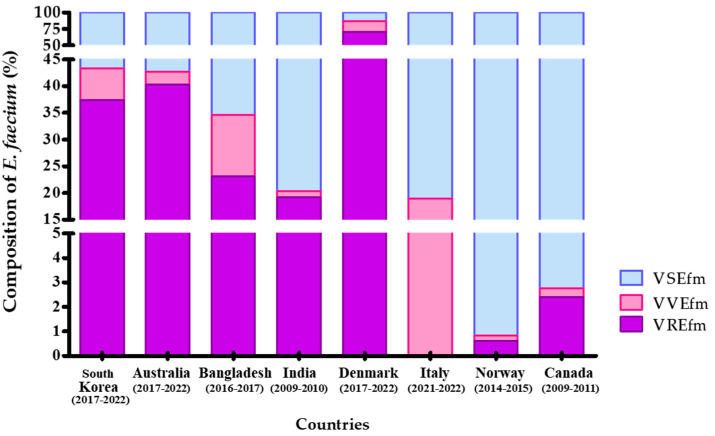
Comparison of Vancomycin-resistant and Vancomycin-variable *Enterococcus faecium* Isolates in the Surveillance System by Countries. In our study, both *vanA/B*-positive and -negative vancomycin-resistant *Enterococcus faecium* (VREfm) isolates were incorporated into the comparative analysis with data from other countries. VREfm, *vanA/B*-positive vancomycin-resistant *E. faecium*; VVEfm, *vanA/B*-positive vancomycin-susceptible *E. faecium*.

VVE isolates have been reported in several countries, including Canada [5], Norway [6], Italy [7], Denmark [17], India [9], Bangladesh [10], and Australia [11]. *E. faecium* isolates in Canada were collected from a single hospital between 2009 and 2011, with 0.4% (52 out of 14,747 isolates) identified as VVEfm (Figure 3). Sivertsen et al. (2016) reported that, in Norway, a total of 15,158 *E. faecium* isolates were collected from hospitals during 2014–2015. Among these, 93 isolates (0.61%) were identified as *vanA* gene-positive *E. faecium*, and 34 of 93 isolates were confirmed to be cases of VVEfm [6]. In Italy, from December 2021 to June 2022, none of the 59 *E. faecium* isolates collected tested positive for VREfm, whereas 11 strains (18.6%) were confirmed as VVEfm [7]. In Denmark, genetic testing was employed instead of antibiotic susceptibility testing. The strains carrying the VAN resistance gene are classified as VVEfm if they belong to sequence type ST1421, while those not identified as ST1421 are classified as VREfm [17]. Of the 4546 *E. faecium* isolates collected between 2017 and 2022, 3972 were identified as VAN resistant (87.4%), and 751 strains were classified as ST1421 VVEfm (16.5%). In India, the data collected through the Indian Council of Medical Research surveillance system between 2019 and 2020 indicate a VVEfm prevalence of 1.2% (5 out of 427 isolates) [9]. In Bangladesh, VVE was assessed in 352 enterococci isolated from farmed animals and fresh retail meats between 2016 and 2017 [10]; among these, three *E. faecium* isolates were classified as VVE (11.5%), and 6 strains out of 26 *E. faecium* were confirmed as VRE. The Australian Group on Antimicrobial Resistance reported the identification of VVEfm in 2.5% (81 isolates) of cases between 2017 and 2022 (https://agargroup.org.au/agar-reports/ (accessed on 1 July 2023)). In research previously conducted in Korea, 7.4% (18 isolates) of *vanA*-type VVEfm were identified in fecal samples from patients at 10 tertiary hospitals between 2004 and 2008 [13]. Similarly, the present study identified 124 *vanA*-type VVE isolates (5.9%) among 2115 *E. faecium* isolates collected through the Kor-GLASS program between 2017 and 2022. According to the studies from Denmark and Australia, conducted through continuous national surveillance systems similar to Korea’s, the proportions of VSEfm, VREfm, and VVEfm in Korea and those in Australia were most similar.

Compared to VVEfm, studies on VVEfs have been reported in only four countries, with findings primarily focused on the presence or absence of the *van* gene. In Norway, both *E. faecium* and *E. faecalis* isolates were collected; however, only a single strain of *vanA*-type VVEfs was identified [6]. Similarly, in Denmark, only two *vanB*-type VVEfs strains were identified out of 659 strains in 2022 [17]. In Bangladesh, 7 out of 105 *E. faecalis* isolates (6.7%), carrying *vanA* or *vanC*, were identified as VVEfs [10]. In Korea, *vanA*-type VVEfs were isolated in 2020 and 2022. Remarkably, in 2021, a sudden surge of VVEfs was observed, with 71 isolates identified, displaying a pattern closely mirroring that of VVEfm. This finding suggests the presence of unique factors in Korea that influence the occurrence and distribution of VVEfm and VVEfs.

### 2.4. Multilocus Sequence Type of VVE in E. faecium and E. faecalis

Clonal complex 17 (CC17) in *E. faecium* is further divided into two major clusters, ST17 and ST817, with ST80 showing a closer phylogenetic relationship to ST817 than ST17. The predominant VVEfm clones in Korea were identified as ST17 (30.6%), ST817 (16.9%), and ST80 (12.9%). Clonal complex 17 (CC17), which includes ST17, ST817, and ST80, accounted for 91.3% of the cases, with no significant changes observed in its dominance. To date, most VVEfm strains are classified as ST1421, ST203, and ST18 [5,6,8,11,17] and are different from ST17, ST817, and ST80, found in this study. For example, the dominant VVEfm clones shifted from ST203 to ST1421 in 2019 in Denmark [17]. In most regions of Australia, all major STs belonged to CC17, a major hospital-adapted polyclonal *E. faecium* cluster (https://agargroup.org.au/agar-reports/ (accessed on 1 July 2023)). In 2022, 85.5% of major STs were ST17, ST78, ST80, ST117, ST555, ST796, ST1421, and ST1424. VVEfm was identified as ST18 in Canada [5], while ST203 was detected during an outbreak in Norway [6]. Conversely, a Japanese study characterized 19 isolates of VVEfm [19], all of which were identified as ST78 with highly related profiles from pulsed-field gel electrophoresis. Interestingly, in Italy, VVEfm isolates were confirmed as ST1478 (45.5%), ST80 (36.3%), ST117 (9.1%), and ST789 (9.1%) [7]. VVE strains have exhibited issues with the *pstS* gene, showing a lack in ST1421 and a null sequence in ST1478 [7]. In Korea, ST1421 was found in 19.9% of VREfm isolates from 2017 to 2020; however, it was not detected in VVEfm isolates until 2022. Kim et al. (2024) also reported that ST1421, characterized by a *pstS* deletion, was confirmed in 43.3% of *vanA* gene-positive VREfm in 2019, and identified VVEfm isolates belonging to three ST17 strains and one ST192 strain [20]. In summary, while Korea has maintained a distinct sequence type for VVEfm, it remains essential to closely monitor the potential shifts in sequence types through the *pstS* gene.

Among the 73 VVEfs isolates, one was detected in 2020, 71 were collected in 2021, and one was detected in 2022. ST16 was detected in 2020, and ST428 was detected in 2022. In 2021, VVEfs exhibited an unusually high incidence with a diverse range of sequence types, predominantly ST179 (23.9%), ST16 (14.1%), and ST28 (9.9%). In contrast to VVEfm, there are no reports on the sequence types of VVEfs. Future studies on VVEfs are necessary to generate more comprehensive data.

## 3. Materials and Methods

### 3.1. Bacterial Collection and Antimicrobial Susceptibility Test

From 2017 to 2022, enterococci were isolated from the bloodstream samples from 9 tertiary general hospitals through the Kor-GLASS program. Also, redundant isolates were excluded from each bloodstream sample. Enterococcus species were identified using matrix-assisted laser desorption ionization-time of flight mass spectrometry (MALDI-TOF MS; Microflex Bruker, Bremen, Germany). Antimicrobial susceptibility testing (AST) for AMP, CIP, HL-GEN, HL-STR, LIN, TET, TEI, TIG, DAP, and VAN was performed according to the Clinical and Laboratory Standards Institute (CLSI) guidelines, with minimal inhibitory concentrations (MICs) determined using the broth microdilution method [21]. The quality control strains, *E. faecalis* ATCC 29212 (wild type), Staphylococcus aureus ATCC 29213 (wild type), and *E. faecalis* ATCC 51299 (*vanB*^+^ VRE), were used. The results were interpreted based on the CLSI (2023) breakpoints [21]. For TIG, where CLSI breakpoints were unavailable, the criteria from the European Committee on Antimicrobial Susceptibility Testing (EUCAST, 2023) were applied [22]. The AST experiment was repeated at least five times with the same antibiotics.

### 3.2. vanA/B Gene PCR and Multilocus Sequence Typing (MLST)

All of the VVE isolates were tested for *vanA* and *vanB* genes and MLST using PCR. Extraction of bacterial DNA was performed using DaBead™ Genomic DNA Prep Kit (BIOFACT, Daejeon-si, Republic of Korea). The presence of *vanA* and *vanB* encoding proteins involved in VAN resistance was determined using multiplex conventional PCR [23]. To confirm *vanA* and *vanB* genes, *E. faecalis* ATCC 29212 served as the negative control, while *E. faecium* ATCC 700221 (*vanA* gene positive VRE) and *E. faecalis* ATCC 51299 (*vanB* gene positive VRE) were used as positive controls. PCR products were resolved by electrophoresis on a 1.5% agarose gel containing SYBR Green, and the *vanA* (377 bp) and *vanB* (298 bp) genes were identified based on their specific sizes.

For MLST, PCR was conducted using seven housekeeping genes (*adk*, *atpA*, *ddl*, *gdh*, *gyd*, *purK*, and *pstS* for *E. faecium*, and *gdh*, *gyd*, *gki*, *aroE*, *xpt*, and *yqiL* for *E. faecalis*) [24,25]. The nucleotide sequences were accurately determined using the Sanger sequencing method with bidirectional analysis. Allele numbers were assigned by comparing the sequence at each locus to the known alleles in the Enterococcus MLST database [26]. The maximum-likelihood phylogenetic trees were constructed using MEGA software version 11.0.13.

### 3.3. VVE Reversion Rate

In order to assess the reversion of VVEfm and VVEfs strains to the resistance phenotypic under laboratory conditions, we tested strains by plating on brain heart infusion (BHI) agar supplemented with increasing concentrations of vancomycin (to 8 μg/mL). The plates were incubated at 37 °C for 48 h, and the growth of VVE strains was counted. The reversion rate was calculated as the ratio of resistant strains to the total VVE population.

### 3.4. Statistical Analysis

The AST results of enterococci isolates were categorized as either susceptible (including intermediate) or resistant and assigned a binary value for each response, with 1 for resistant and 0 for susceptible. One-way ANOVA with LSD post-hoc test was used to compare antibiotic resistance rates of enterococci by year. The intended level of the tests is set at 0.05. All data were statistically analyzed using IBM SPSS Statistics version 29.0 (IBM Corp, Armonk, NY, USA).

## 4. Conclusions

This study is the first to report the prevalence of VVE at multicenter, regional, and national levels in Korea. Through the Kor-GLASS program, we observed a rapid increase in *vanA*-type VVEs following the COVID-19 pandemic in 2020. While *vanA*-type VREs showed a slight decline, VVE cases continued to rise. Our classification of VREs is predicated on the presence of the *vanA* gene and VAN resistance. If we had relied exclusively on phenotypic testing, we would have failed to detect VVE. Although this study relied solely on confirming the vancomycin resistance genes *vanA* and *vanB*, which may have led to an underestimation of VVE prevalence, this observation underscores the importance of molecular testing for *van* genes to ensure accurate detection and effective surveillance. The increased use of antibiotics among COVID-19 patients may have contributed to this trend by promoting the expression of the *van* genes. Although VRE cases decreased in 2022, effective infection control remains essential, such as isolating patients diagnosed with VVE in the same manner as VRE. We plan to continue utilizing the Kor-GLASS program to monitor this trend and assess potential resistance to key VRE treatment antibiotics, including linezolid, tigecycline, and daptomycin.

## Figures and Tables

**Table 1:** Antimicrobial Susceptibility and Resistance Trends of VSEfm, VVEfm, and VREfm.

*Enterococcus faecium* (n = 2115)	Years	Antibiotic Resistant Rate (%)									
		AMP	CIP	HL-GEN	HL-STR	TET	TEI	LIN	TIG	DAP	QDA
VSEfm (n = 1199)	2017 (n = 186)	83.9	84.4	18.3	1.6	17.7	0	0	0.5	-	3.2
	2018 (n = 167)	85.6	84.4	16.2	0	16.8	0	0	0	-	6.0
	2019 (n = 182)	83.5	84.1	12.1	1.1	15.4	0	0	0.5	-	9.3
	2020 (n = 208)	78.8	80.8	17.3	2.9	10.1	0	0	0	0	3.8
	2021 (n = 175)	82.9	87.4	24.0	4.6	15.4	0	0	1.1	3.4	25.7
	2022 (n = 281)	82.9	88.3	17.8	6.4	12.5	0	0	0	0.4	21.7
VVEfm (n = 124)	2017 (n = 4)	100	100	0	0	25.0	0	0	0	-	0
	2018 (n = 1)	100	100	0	0	0	0	0	0	-	0
	2019 (n = 0)	-	-	-	-	-	-	-	-	-	-
	2020 (n = 10)	90.0	90.0	70.0	10.0	0	0	0	0	0	10.0
	2021 (n = 78)	76.9	84.6	15.4	5.1	9.0	0	0	0	5.1	25.6
	2022 (n = 31)	90.3	93.5	29.0	6.5	12.9	0	0	0	0	12.9
VREfm (n = 792)	2017 (n = 98)	100	100	18.4	0	9.2	55.1	0	0	-	3.1
	2018 (n = 109)	99.1	99.1	29.4	0	14.7	68.8	0	0	-	6.4
	2019 (n = 126)	100	100	40.5	0	7.1	84.9	0	0	-	7.1
	2020 (n = 137)	97.1	97.8	40.9	1.5	7.3	88.1	0	0	0	5.1
	2021 (n = 153)	100	100	25.5	3.9	9.2	37.9	0	0	2.0	52.9
	2022 (n = 169)	99.4	100	42.0	1.8	16.0	68.6	0	0.6	0.6	24.3

Numbers in parentheses represent non-susceptible antibiotic rates. AMP, ampicillin; CIP, ciprofloxacin; DAP, daptomycin; HL-GEN, high-level gentamycin; HL-STR, high-level streptomycin; LIN, linezolid; n, number; QDA, quinupristin/dalfopristin; TET, tetracycline; TEI, teicoplanin; TIG, tigecycline; VSEfm, vancomycin-susceptible *E. faecium*; VREfm, vancomycin resistance *E. faecium* (vanA/B+ VREfm and vanA/B− VREfm); VVEfm, *vanA/B* gene-positive vancomycin-susceptible *E. faecium*; -, not tested.

## Data Availability

All data generated or analyzed during this study are included in this published article and its Supplementary Materials.

## Supplementary Materials

The following supporting information can be downloaded at https://www.mdpi.com/article/10.3390/antibiotics13121210/s1. Figure S1: A Maximum-Likelihood Tree; Table S1 Antimicrobial Susceptibility and Resistance Trends of VSEfs, VVEfs, and VREfs.
