# Supplementary material for: Prevalence of Vancomycin-Variable Enterococci from the Bloodstream in the Korea Global Antibiotic Resistance Surveillance System, 2017–2022
Source: Antibiotics (Basel). 2024 Dec 12;13(12):1210. doi: 10.3390/antibiotics13121210 (PMC11672432; doi:10.3390/antibiotics13121210)
Supplement: Supplementary file 1 [file antibiotics-13-01210-s001.zip › antibiotics-3323718-supplementary.pdf]

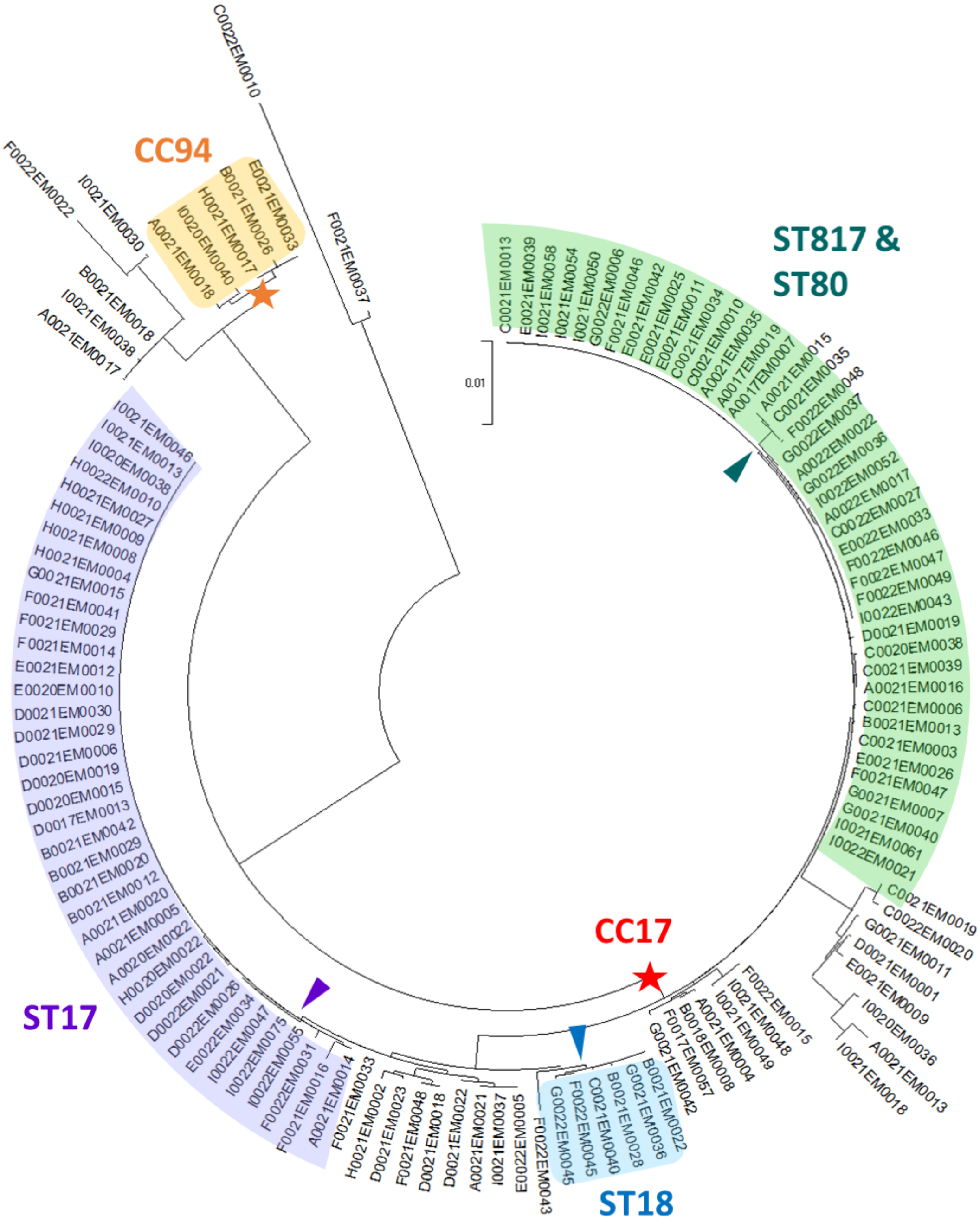

Supplementary Figure S1. A Maximum-Likelihood Tree.

The tree was constructed using MLST to analyse VVEfm cases isolated from bloodstream samples across nine tertiary general hospitals. The tree was generated using the Kimura 2-parameter model. The shaded areas indicate clusters corresponding to the predominant STs.

Supplementary Table S1. Antimicrobial Susceptibility and Resistance Trends of VSEfs, VVEfs, and VREfs

Enterococcus faecalis **(n = 1,227)**	**Years**	**Antibiotic Resistance Rate (%)**									
		**AMP**	**CIP**	**HL-GEN**	**HL-STR**	**TET**	**TEI**	**LIN**	**TIG**	**DAP**	**QDA**
**VSEfs (n = 1,143)**	2017 (n = 175)	0.6	40.6	26.3	5.7	73.1	0	1.1	0	-	-
	2018 (n = 178)	1.7	39.3	29.2	5.6	74.7	0	0	0	-	-
	2019 (n = 178)	5.1	34.3	22.5	6.7	68.5	0	0.6	0	-	-
	2020 (n = 201)	8.5	30.8	37.3	10.9	69.7	0.5	0	0	0	-
	2021 (n = 171)	0	27.5	30.4	8.2	66.1	0	0	0	0	-
	2022 (n = 240)	0	30.8	37.9	10.4	70.8	0	0	0	0	-
**VVEfs (n = 73)**	2017 (n = 0)	-	-	-	-	-	-	-	-	-	-
	2018 (n = 0)	-	-	-	-	-	-	-	-	-	-
	2019 (n = 0)	-	-	-	-	-	-	-	-	-	-
	2020 (n = 1)	0	0	100	100	100	0	0	0	0	-
	2021 (n = 71)	0	28.2	38.0	2.8	63.4	0	0	0	0	-
	2022 (n = 1)	0	0	0	0	0	0	0	0	0	-
**VREfs (n = 11)**	2017 (n = 1)	0	100	100	100	100	100	0	0	-	-
	2018 (n = 1)	0	100	100	0	100	100	0	0	-	-
	2019 (n = 3)	33.3	100	0	0	66.7	100	0	0	-	-
	2020 (n = 2)	100	100	100	0	100	100	0	0	0	-
	2021 (n = 1)	0	100	0	0	100	100	0	0	0	-
	2022 (n = 3)	0	100	100	0	100	66.7	0	0	0	-

-: not tested.

Numbers in parentheses mean non-susceptible antibiotic rates. AMP, ampicillin; CIP, ciprofloxacin; HL-GEN, high-level gentamycin; HL-STR, high-level streptomycin; DAP, daptomycin; LIN, linezolid; n, number; QDA, quinupristin/dalfopristin; TEI, teicoplanin; TET, tetracycline; TIG, tigecycline; VSEfs, vancomycin-susceptible *E. faecalis*; VREfs, vancomycin resistance *E. faecalis* (vanA/B+ VREfs and vanA/B− VREfs); VVEfs, *vanA/B* gene-positive vancomycin-susceptible *E. faecalis*.
